# Novel Dengue Virus Type 1 from Travelers to Yap State, Micronesia

**DOI:** 10.3201/eid1202.050733

**Published:** 2006-02

**Authors:** Yoko Nukui, Shigeru Tajima, Akira Kotaki, Mikako Ito, Tomohiko Takasaki, Kazuhiko Koike, Ichiro Kurane

**Affiliations:** * National Institute of Infectious Diseases, Tokyo, Japan;; † University of Tokyo, Tokyo, Japan

**Keywords:** Dengue, sequence deletion, untranslated region, dispatch

## Abstract

Dengue virus type 1 (DENV-1), which was responsible for the dengue fever outbreak in Yap State, Micronesia, in 2004, was isolated from serum samples of 4 dengue patients in Japan. Genome sequencing demonstrated that this virus belonged to genotype IV and had a 29-nucleotide deletion in the 3´ noncoding region.

Dengue virus (DENV) is a mosquitoborne flaviviruses; there are 4 serotypes, DENV-1, -2, -3 and -4. DENV has been found in >100 countries and 2.5 billion people live in areas where dengue is endemic. Fifty to one hundred million cases of dengue infection are estimated to occur annually (*1*). In Japan, outbreaks of dengue fever occurred in Nagasaki, Hiroshima, Kobe, and Osaka from 1942 to 1945, but none thereafter (*2*). However, ≈50 imported dengue cases occur annually in Japan.

The DENV genome is a single-stranded positive-sense RNA of ≈11,000 nucleotides (nt) that encodes 3 structural proteins (capsid, membrane, and envelope) and 7 nonstructural proteins (NS1, NS2A, NS2B, NS3, NS4A, NS4B, and NS5) (*3*). Surrounding the open reading frame (ORF) are 5´ and 3´ noncoding regions (NCRs) that form RNA secondary structures (*4*). These regions are ≈100 and ≈400 nt, respectively (*5*).

Dengue fever developed in 9 Japanese patients in 2004 after they returned from Yap state. We report the genetic characterization of RNA from DENV-1 isolates from these patients.

## The Study

Yap is the westernmost state of the Federated States of Micronesia and composed of 4 major islands. Yap has a total area of 102 km^2^ and a population of 11,241 (2000 census). The climate is moderate and fairly constant. The mean annual temperature is 27°C. Relative humidity ranges from 65% to 100% (annual mean 83%). Rainfall averages 120 inches a year and is seasonal.

In 1995, a dengue epidemic caused by DENV-4 occurred in Yap state (*6*), but no dengue outbreaks have since been reported. However, the Yap Epinet Team reported a dengue outbreak caused by DENV-1 in Yap state that began in the last week of May 2004. A total of 658 reported dengue fever cases (defined by the World Health Organization) occurred as of December 29, 2004. No deaths or dengue hemorrhagic fever/dengue shock syndrome cases were reported (*7*).

Fever, headache, and diarrhea developed in 7 Japanese adults who visited Yap after their return to Japan in August 2004. DENV infection was serologically confirmed in 5 patients (patients 1–5) by an immunoglobulin M (IgM) capture enzyme-linked immunosorbent assay (ELISA) (Focus Diagnostics Inc., Herndon, VA, USA) and an IgG ELISA (PANBIO Ltd., Brisbane, Queensland, Australia) at the National Institute of Infectious Diseases in Tokyo, Japan. Of these 5 patients, 4 had a primary DENV infection and 1 had a primary dengue infection and a secondary flavivirus infection. DENV infection was serologically confirmed in the sixth patient at another institute. The seventh patient did not visit a medical facility but had symptoms of dengue fever. In addition, 2 other Japanese patients who traveled to Yap in September 2004 were diagnosed with dengue (data not shown).

Four virus isolates (NIID04-27, -31, -41, and -47) were obtained from serum samples from patients 1–4, respectively. Two hundred microliters of serum samples diluted 1:40 was injected onto C6/36 cells in minimal essential medium supplemented with 2% fetal calf serum. The cells were incubated at 28°C for 7 days and culture supernatant fluids were collected. DENV isolates were used for analysis without any further passage.

Complete nucleotide sequencing of RNA of NIID04-27, -31, and -47 and partial sequencing of NIID04-41 were performed. Viral RNA was extracted by using a High Pure RNA extraction kit (Roche Diagnostics, Mannheim, Germany) according to the manufacturer's instructions, transcribed to cDNA, and amplified by polymerase chain reaction, as described previously (*8*). The cDNA was purified and sequenced by using the ABI PRISM 3100 Avant Genetic Analyzer (Applied Biosystems, Foster City, CA, USA). Seventeen pairs of primers were designed based on the DENV-1 NIID02-20 sequence (GenBank accession no. AB178040) and used in the analyses (*9*).

The nucleotide sequences of the viral isolates were compared with published complete sequences of DENV-1 (Table 1). Sequence alignment and analysis were performed by using ATGC analysis programs (version 4.02; Genetyx Corp., Tokyo, Japan). Phylogenetic analyses of nucleotide sequences were conducted with ClustalX software version 1.83 (ftp://ftp-igbmc.u-strasbg.fr/pub/ClusterW/). A phylogenetic tree was reconstructed for aligned nucleotide sequences by using the neighbor-joining method. Bootstrap reassembling analysis of 1,000 replicates was used to assess confidence values for virus groupings. The phylogenetic tree was constructed by using Treeview software version 1.6.6 (http://taxonomy.zoology.gla.ac.uk/rod/treeview.htm).

**Table 1:** Dengue virus (DENV) strains used in the study

Virus	Strain	Origin	Year isolated	Genbank accession no.
DENV-1	NIID04-27	Yap Island	2004	AB204803
DENV-1	NIID03-41	Republic of Seychelles	2003	AB195673
DENV-1	FGA/89	French Guiana	1989	AF226687
DENV-1	BR/90	Brazil	1990	AF226685
DENV-1	BR/97-111	Brazil	1997	AF311956
DENV-1	BR/01-MR	Brazil	2001	AF513110
DENV-1	Abidjan	Côte d'Ivoire	1998	AF298807
DENV-1	Mochizuki	Japan	1943	AB074760
DENV-1	S275/90	Singapore	1990	M87512
DENV-1	16007	Thailand	1964	AF180817
DENV-1	GZ/80	China	1980	AF350498
DENV-1	A88	Indonesia	1988	AB074761
DENV-1	Cambodia	Cambodia	1998	AF309641
DENV-1	Djibouti	Ethiopia	1998	AF298808
DENV-1	West Pac 74	Nauru	1974	U88535
DENV-1	98901530	Indonesia	1998	AB189121
DENV-1	98901518	Indonesia	1998	AB189120
DENV-1	259par00	Paraguay	2000	AF514883
DENV-1	295arg00	Argentina	2000	AF514885
DENV-1	ARG9920	Argentina	1999	AY277664
DENV-1	NIID02-20	Thailand	2002	AB178040
DENV-1	99-36-1HuNIID	Paraguay	1999	AB111065
DENV-1	01-27-1HuNIID	The Philippines	2001	–
DENV-1	01-32-1HuNIID	The Philippines	2001	–
DENV-1	01-36-1HuNIID	Singapore, Malaysia	2001	AB111067
DENV-1	01-42-1HuNIID	Thailand, Cambodia	2001	AB111069
DENV-1	01-44-1HuNIID	Tahiti	2001	AB111070
DENV-1	01-54-1HuNIID	India	2001	–
DENV-1	01-54b-1HuNIID	India	2001	–
DENV-1	01-61-1HuNIID	Cambodia	2001	AB111071
DENV-1	01-65-1HuNIID	Thailand	2001	AB111072
DENV-1	01-66-1HuNIID	Thailand	2001	–
DENV-2	DENtype2-TB16i	Indonesia	2004	AY858036
DENV-3	DENtype3-TB55i	Indonesia	2004	AY858048
DENV-4	DENtype4-8976/95	Indonesia	2004	AY762085

The full-length RNA genomes of NIID04-27, -31, and -47 were 10,706 nt. A previous study reported that the full-length RNA genome of DENV-1 was 10,735 nt (*8*). The differences in the genome sequence between NIID04-27 and the other 2 isolates (NIID04-31 and -47) were subtle; identities with NIID04-31 and -47 were 99.94% and 99.92%, respectively. The results suggest that these 3 isolates belong to the same strain. Therefore, we used NIID04-27 as a representative isolate for further analysis.

**Figure 1:**
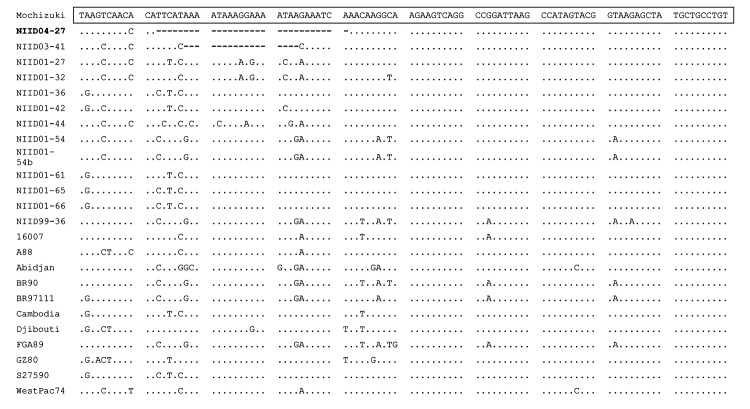
Nucleotide sequence alignment of the variable region in the 3´ noncoding region of dengue virus type 1 strains, including NIID04-27 sequenced in the present study. The Mochizuki strain was used as the consensus sequence, and the sequence of 100 nucleotides immediately downstream of the open reading frame termination codon is shown at the top. Solid dots indicate nucleotides identical to the consensus sequence and hyphens indicate deletions.

To characterize the molecular structure of the genome, the complete NIID04-27 nucleotide sequence was compared with those of other DENV-1 strains available in GenBank (Table 1). NIID04-27 shared sequence identity ranging from 90.9% to 96.9% (Table 2) with 12 other DENV-1 strains. With respect to the alignment of full-length genomes, some alterations were found in the 3´ NCR. These alterations included a deletion of 29 nt starting at the 13th position from the ORF termination codon (Figure 1). The same deletion in the 3´ NCR was found in the viral genome amplified directly from the serum sample from patient 1 and was also observed in NIID04-31, -41, and -47.

**Table 2:** Pairwise comparisons of full-length genome and 3´ noncoding region sequences of dengue virus type 1 (DENV-1) strains*

Virus strain	% identify of nucleotide												
	NIID04-27	FGA/89	BR/90	BR/97-111	Abidjan	Mochizuki	S275/90	16007	GZ/80	A88	Cambodia	Djibouti	WestPac74
	Full-length genome												
NIID04-27		**91.5**	**91.5**	**91.3**	**90.9**	**93.4**	**91.9**	**93.1**	**92.3**	**96.9**	**91.7**	**91.7**	**95.6**
FGA/89	**89.5**		98.3	97.9	94.5	93.7	93.4	93.5	92.5	92.3	92.0	92.1	93.3
BR/90	**89.5**	99.4		98.8	94.5	93.9	93.7	93.7	92.7	92.3	92.2	92.0	92.9
BR/97-111	**89.3**	98.7	99.4		94.4	93.7	93.5	93.5	92.5	92.2	92.1	91.8	92.8
Abidjan	**89.7**	94.0	93.8	94.2		92.9	92.8	92.9	92.1	92.0	91.7	91.6	92.3
Mochizuki	**91.2**	94.8	94.7	94.9	93.4		95.0	95.4	96.1	94.6	95.3	95.2	95.1
S275/90	**91.2**	91.7	91.5	91.9	96.6	94.4		93.8	96.1	93.0	96.4	95.2	93.7
16007	**90.8**	96.2	95.9	95.7	93.2	97.4	93.4		94.2	94.2	93.7	93.5	94.7
GZ/80	**90.2**	94.1	93.8	94.4	93.6	97.4	94.4	95.7		93.3	96.7	98.0	93.9
A88	**92.5**	93.8	93.6	93.8	92.3	96.8	92.9	96.8	95.5		92.7	92.7	97.2
Cambodia	**89.9**	92.1	92.1	92.1	96.4	94.4	96.9	93.6	95.1	92.8		95.9	93.4
Djibouti	**89.5**	92.1	91.9	91.9	95.3	95.3	96.9	94.0	95.5	93.2	97.6		93.3
WestPac74	**91.4**	95.1	94.9	95.1	93.8	93.8	93.2	97.2	95.1	97.2	93.0	93.4	
	3´ noncoding region												

*The percentage nucleotide sequence identities of the complete genomes are shown in the upper right half of the table. The percentage nucleotide sequence identities of the 3´ noncoding region of the genomes are shown in the lower left half of the table. The percentage sequence homologies between NIID04-27 and each of 12 other DENV-1 strains are shown in **boldface**.

To further analyze the genetic variation in the 3´ NCR of DENV-1, we analyzed the sequence of 24 other DENV-1 strains. Only the NIID03-41 strain, which was isolated in our laboratory from a patient returning from the Republic of Seychelles, had a 17-nt deletion in the 3´ NCR (Figure 1). The complete genomes of the 25 DENV-1 strains analyzed showed high levels of nucleotide sequence identity in the 3´ NCR, except for a small region of 50 nt immediately after the ORF, which is the hypervariable region. The nucleotide sequence identities in the 3´ NCR between NIID04-27 and 12 other DENV-1 strains ranged from 89.3% to 92.5% (Table 2).

**Figure 2:**
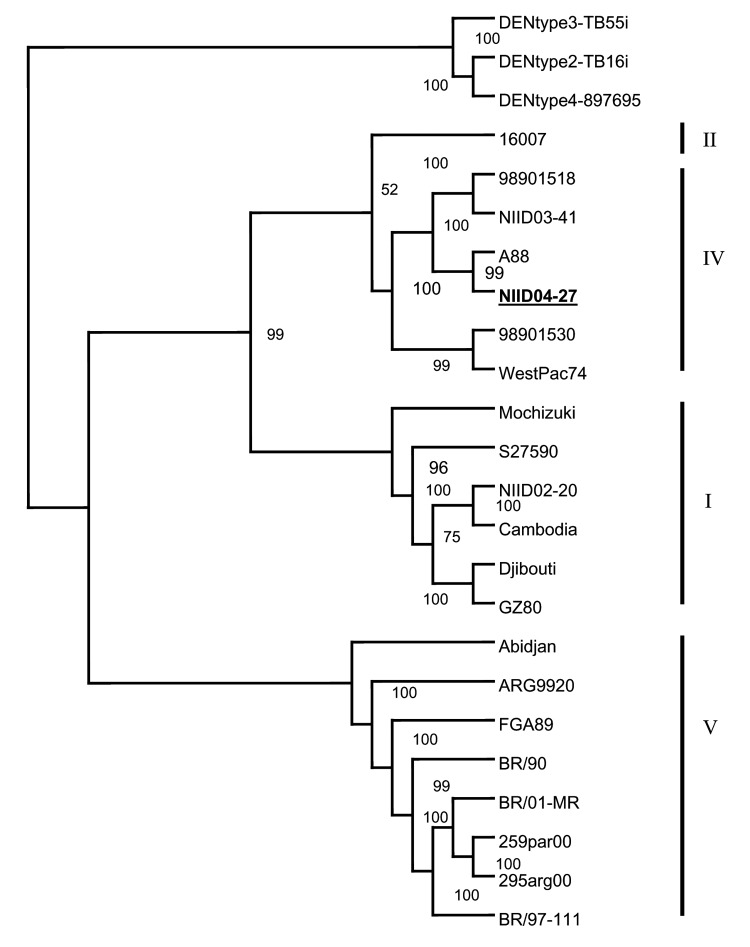
Phylogenetic tree based on the full-length genome sequence of 21 available dengue virus (DENV) type 1 strains and DENV-2, -3, and -4. The multiple sequence alignments were obtained with ClustalX, and the tree was constructed by the neighbor-joining method. The percentage of successful bootstrap replicates is indicated at the nodes. The NIID04-27 strain is indicated in **boldface**. Genotypes I, II, IV, and V correspond to DENV-1 genotypes as defined by Goncalvez et al. (*10*).

To understand the genetic relationships and evolution of DENV-1 strains, we also performed phylogenetic analysis of the fully sequenced DENV-1 strains that included NIID04-27 (Figure 2). NIID 04-27 belonged to genotype IV along with A88, 98901518, 98901530, NIID03-41 and West Pac74 .This cluster was called the Pacific group in a previous report (*10*). NIID04-27 and NIID03-41 are the first DENV-1 strains to have deletions in 3´ NCR.

## Conclusions

We have genetically characterized DENV-1 isolate NIID04-27 by determining its complete nucleotide sequence and comparing the sequence with most of the available DENV-1 full-length sequences. Sequence heterogeneity in the 3´ NCR of the genus *Flavivirus* has been reported for tickborne encephalitis virus, Japanese encephalitis virus, DENV-2, and DENV-4 (*11**–**13*). For example, DENV-2 isolated in Texas, Peru, Venezuela, Mexico, and Puerto Rico had a 10-nt deletion starting at the 19th nucleotide position from the ORF termination codon (*13*).

The terminus of the 3´ NCR has a conserved sequence and secondary structure. The functions of the 3´ NCR of flaviviruses have not been fully determined. The 3´ NCR in flaviviruses affects RNA replication but does not affect viral translation (*14**,**15*). Introduction of a 30-nt deletion starting at the 212th position from the ORF termination codon in the 3´ NCR of DENV-4 reduced the ability of the virus to propagate in vivo and in vitro (*16*).

We have identified a 29-nt deletion in the 3´ NCR of DENV-1 isolated from a dengue patient returning to Japan from Yap. Isolates from 3 other patients infected in the same outbreak also had the same deletion. The DENV-1 strain with a 29-nt deletion in the 3´ NCR was responsible for the dengue epidemic in Yap in 2004. The biologic characteristics induced by this deletion should be further analyzed.
